# Grounded reality meets machine learning: A deep-narrative analysis framework for energy policy research

**DOI:** 10.1016/j.erss.2020.101704

**Published:** 2020-11

**Authors:** Ramit Debnath, Sarah Darby, Ronita Bardhan, Kamiar Mohaddes, Minna Sunikka-Blank

**Affiliations:** a Behaviour and Building Performance Group, The Martin Centre for Architectural and Urban Studies, Department of Architecture, University of Cambridge, Cambridge CB2 1PX, United Kingdom; b Energy Policy Research Group, Judge Business School, University of Cambridge, Cambridge CB2 1AG, United Kingdom; c Energy Program, Environmental Change Institute, University of Oxford, Oxford OX1 3QY, United Kingdom; d Judge Business School, University of Cambridge, CB2 1AG, United Kingdom

**Keywords:** Energy policy, Narratives, Topic modelling, Computational social science, Text analysis, Methodological framework

## Abstract

Text-based data sources like narratives and stories have become increasingly popular as critical insight generator in energy research and social science. However, their implications in policy application usually remain superficial and fail to fully exploit state-of-the-art resources which digital era holds for text analysis. This paper illustrates the potential of deep-narrative analysis in energy policy research using text analysis tools from the cutting-edge domain of computational social sciences, notably topic modelling. We argue that a nested application of topic modelling and grounded theory in narrative analysis promises advances in areas where manual-coding driven narrative analysis has traditionally struggled with directionality biases, scaling, systematisation and repeatability. The nested application of the topic model and the grounded theory goes beyond the frequentist approach of narrative analysis and introduces insight generation capabilities based on the probability distribution of words and topics in a text corpus. In this manner, our proposed methodology deconstructs the corpus and enables the analyst to answer research questions based on the foundational element of the text data structure. We verify theoretical compatibility through a meta-analysis of a state-of-the-art bibliographic database on energy policy, narratives and computational social science. Furthermore, we establish a proof-of-concept using a narrative-based case study on energy externalities in slum rehabilitation housing in Mumbai, India. We find that the nested application contributes to the literature gap on the need for multidisciplinary methodologies that can systematically include qualitative evidence into policymaking.

## 1 Introduction

The energy policymaking needs in today’s world cannot be met alone by the existing methods of quantification due to the multi-agent nature of energy justice and its associated challenges [1]. Clearer representation of these deep interdependencies is well-captured by the United Nation’s 17 Sustainable Development Goals (SDGs) that provides a bird’s eye view of the global policy and technology challenges. An argument often asserted by policymakers and scientists is that, despite ‘sound’ technology and ‘rational’ policies, policy implementation yields unexpected results. It is often due to the lack of multidisciplinary insights into the energy policymaking and implementation processes [1].

The current regime of energy policymaking by scientists and technologists is guided by the promotion of preferred technologies with theoretical properties that complements the objectives of the leading political forces. They justify their proposition based on quantitative models of energy and climate systems that graft directional biases in the policymaking, often overlooking the strengths of qualitative stakeholder deliberations. ‘Good’ energy policy under a technocratic directional bias thus becomes policy focussed on ‘getting the technology right’ [1]. While technology can indeed be a force to achieve climate change mitigation and sustainability goals, its intended welfare effects may be restricted if the wants of a society are not appropriately reflected in policy [2]. Non-economic drivers may be crucial in achieving the welfare effects from specific energy and climate policy, and they remain underrepresented in the current literature. So much so that, even the work of the Intergovernmental Panel on Climate Change (IPCC) has been characterised as significantly directional and ‘unidisciplinary’ as it is based on a clear separation between the natural sciences and social sciences, and an understanding that social sciences are based on natural sciences [3]. The problem with such technocratic directionality bias is that it leads to an inherent limitation in the definition of policy goals, which become defined as ‘reasonable’ or ‘rational’ according to technical parameters.

Purely technical approaches cannot account for the multidimensional nature of the socio-cultural forces that effects policy implementation. Oversimplification of policies through quantitative and frequentist generalisation of contexts remain energy policymaking challenges. Therefore, Ozawa et al. [1], state that the premise of ‘good’ energy policy is much more multi-layered, nuanced and non-obvious. Directional bias among policymakers and scientists misses this premise. A multidisciplinary perspective to modern-day energy policymaking requires a deep understanding of historical and of social, political and cultural institutions that constitute the people, places and practices. It demands serious inclusion of social science and humanities thinking in energy policy, thereby leading to a much richer set of insights for ‘good’ energy policy in the 21st Century. However, an inherent problem with the qualitative approaches of social science and humanities for policy applications remain with the directionality of its application. It can cause inferential biases that lead to issues of reliability, validity, replicability and generalisability in qualitative research [4].

A critical and relatively new object of energy and climate change research through the lens of social science and humanities is the use of narratives as a way of crystallising arguments and assumptions [5]. Narratives, stories and storytelling have garnered credible representation in energy and climate change research and policy [6], [7], [8], [9], [10]. However, the value of these narratives depends significantly on its interpretation by researchers and practitioners which can induce biases in the interpretability of the results [5]. Some authors claim that such biases make narratives-driven methods ineffectual as compared to science and technology-driven quantitative methods [11], [12]; though, it is worth bearing in mind that methods based on the ‘hard’ sciences will also have inbuilt empirical biases, for example in the choice of metrics or indicators. These methods are also likely to miss nuances in the use of language and lose some of the layered meanings intended by narrators. On the contrary, a human who is fully conversant with the language used can capture the contextual socio-cultural gradations by using robust qualitative methodological tools like narrative analysis using grounded theory.

The primary aim of this paper is to improve the robustness of narrative analysis in public policy research. Here, we focus exclusively on energy policy applications. In doing so, we propose a nested methodological framework based on current state-of-the-art techniques of data collection, qualitative analysis and computational social sciences. The novel nested approach presented here contributes to lessening directional biases in narrative-driven policy analysis. The innovation of this study lies in the nested treatment of two epistemologically parallel but methodologically distinct concepts [13] of topic modelling (TM) and grounded theory (GT). These parallel methods are used as complementary support to each other which can reduce directional bias in energy policy applications.

By improving the robustness, we want to expand the applicability of text-based tools like narratives, stories and storytelling into the disciplines of evidence-based policymaking [14]. While the experience of a qualitative researcher remains the most critical control variable for narrative-driven analysis, manual coding of texts is highly taxing and is limited by human cognitive abilities. At best, it allows for sensitive and insightful analysis. There is always the likelihood of some directional and researchers’ bias in the complex narrative analysis [15]. We enrich the manual-coding processes of narrative analysis by employing artificial intelligence (AI) and machine-learning aided topic detection algorithm, i.e., topic modelling (TM), that can if well-designed, lessen directionality biases. Recent studies have shown that qualitative researchers, guided by the principles of grounded theory (GT) [16], [17] have incorporated a combination of manual-coding methodologies like open coding, axial coding and selective coding to improve the robustness of their analysis [15], [18]. Our proposed methodology replaces these steps with a probabilistic step of ‘word’ selection using TM that statistically improves the robustness of results. This step lessens directionality bias as TM deconstructs the text-corpus of the narratives into its fundamental constituent (i.e., words/terms) and aids the analyst to reconstruct the narratives through a GT driven-interpretivist approach.

The proposed deep narrative analysis framework does not intend to replace each of these distinct methods. Instead, it shows the synergistic capability of such methods that contributes to the growing literature of computational social sciences and energy policy research. The central function of the proposed nested methodology is to deconstruct a text corpus collected as narratives and identify patterns among word-word, word-topic and topic-topic probabilities in the corpus. It is intended to remove directionality biases in order to enable an analyst to derive more precise insights from the narratives through grounded theory for policy applications. We establish this claim by investigating its theoretical compatibility using two metrices, epistemological-fit and meta-theoretical fit. The theoretical compatibility assessment was performed through science mapping of relevant literature in the current bibliographical databases. Parallelly, a proof-of-concept was presented through a case study of energy service externalities in poverty in slum rehabilitation housing (SRH) in Mumbai, India. Primary data for the case study was collected through a gendered focus group discussion on the problems of high electricity bills in the SRH built environment.

Deconstruction of the raw-narratives into their fundamental elements (words), and then reconstructing them through an interpretivist GT application not only removes directionality biases, but it also preserves the cognitive essence of a narrative-analysis. To the best of our knowledge, we believe such a nested approach has never been developed or applied to humanities and social science bounded evidence-based energy policy research. Our proposed framework contributes to the growing field of computational social sciences while expanding the applicability of rigorous qualitative methods like GT-based narrative analysis. While in this paper, we develop the nested approach, in [19], we illustrate the application of the proposed methodology for deriving energy justice pathways in the Global South.

## 2 Background

### 2.1 Narrative-analysis in energy policy research

Narratives, stories and storytelling go beyond the analytical convention of energy and climate change research. It helps in approaching the intersection of nature, humanity, and technology in a multidisciplinary manner, using a lens from social sciences, humanities, and practitioners ‘perspectives [8]. A special issue of *Energy Research & Social Science* journal, titled ‘Narratives and Storytelling in Energy and Climate Change Research’ [20] have established the importance of narrative, stories and storytelling as critical data objects in energy and climate change research. This special issue presented 34 articles that employ stories and storytelling as avenues of gathering new data and understanding, communication, and influencing others. Narrative analysis in the same issue presented novel ways of crystallising arguments and assumptions that generated deeper policy insights [5].

The energy and climate policy research is however, young with a few instances of narrative analysis in the published literature. Bushell, Buisson, Workman, & Colley, [21] proposed multiple narrative designs to promote action on climate change and development of strategic-narratives to engage multiple stakeholders better. Hermwille [22] advanced the role of narratives in the crystallisation and structuring of arguments in socio-technical energy policy discourse by citing examples from Fukushima and energy regimes of Japan, Germany and the United Kingdom. In doing so, the author captured the policy responses from multiple-perspectives. It is one of the rare application of narratives to generate socio-technical policy responses. However, as mentioned in Section 1, such application is limited by the directionality and interpretability of the researcher, that can subconsciously induce biases. Hermwille [22] in his concluding remark, stated that there is a need for explorative methodologies that can unveil contemporary mechanisms of people, places and practices. Structured Dialogic Design Processes (SDDP) is another methodology that wishes to address some of the explicit normative biases of narrative-based analysis by using web-based communication platforms, thus leveraging information and communication technologies (ICT) [23]. However, SDDP uses observer-dependent data and entirely depends on engaging stakeholders as “expert observers” of the situation in which they are embedded. While it remains a strength of this method, the reliability of this methodology depends on the ability of the expert observers to take action in their situation [24]. This may lead to directional biases in its generalised interpretation. A methodological advancement to a similar discourse-analysis through topic modelling was proposed by Jacobs & Tschötschel [25] which provides a critical epistemological background for this study.

Methodologically there has not been much development in the use and treatment of narratives in energy and climate change research. Most developments were in the mode of data collection using narratives. For example, Shaw & Corner [26] developed a narrative workshop methodology to engage the public in climate change policies. Their innovation lied in the data collection and narrative generation regimes that enabled the public to validate their values and identity, but the results embedded directionality biases based on the interpretation of the researchers. Smith et al. [27], used the concept of ‘energy utopias’ to create experimental stories and generated narratives to refresh public and political conversations about energy and decarbonisation. They found that stories and narratives by themselves do not drive transformation, but they provide confidence in decision-making and public participation.

Similarly, Howarth [28] found that narratives and stories could increase experiential engagement on climate change. Stories and narratives on policy could fill in the information deficit in the present techno-economic models. Our observations from these state-of-the-art articles was that the narratives and stories provided excellent multi-stakeholder engagement platforms, but they offered piecemeal solution to the problem. The methods discussed above have their basis in grounded theory (GT), as each aims to derive a situational-theory based on the narrative-analysis. Thus, we used GT as a foundational component of the nested-model to retain the interpretive effectiveness of narrative-analysis in a computational environment.

Our proposed nested model is based on the foundation of a probability distribution that provides a higher degree of freedom to *zoom-in*, *zoom-out* and *zoom-through* the problem. With zooming-out, possibilities and assumptions that are forgotten or are taken for granted can be better recognised; zooming-in aids better understanding of the granularity that is hidden in the frequentist summaries of central tendencies, like granularities hidden in often-repeated statements. Most importantly, the ability to zoom-through in a computational model can aid in critically analysing narratives as to material reality, norms and practices that determine the energy culture. These three functions cumulatively illustrate our claim of a ‘deep-narrative’ analysis using unsupervised machine learning techniques.

### 2.2 Topic modelling in humanities and social science research

Computational text analysis is an emerging critical methodology in the field of social science and humanities that represents a part of the state-of-the-art in computational social sciences. It centres around probabilistic topic modelling that distributes vocabulary over probability distribution. The high probability words in each distribution can be readily interpreted as recognizable themes, and are thus referred to as “topics” [13], [29], [30]. There are many methods of topic modelling, of which Latent Dirichlet Allocation (LDA) is a widely used method.

LDA is an advanced textual analysis technique grounded in computational linguistics research that calculates the statistical correlations among words in a large set of documents to identify and quantify the underlying (latent) topics in these documents. In linguistic science, Vulić, De Smet, & Moens [31] have used LDA to identify the translation of words among languages. Bauer, Noulas, Seaghdha, Clark, & Mascolo, [32] have used topic modelling to analyse millions of textual comments of geographical and temporal check-ins to understand behavioural pattern at a global scale. Lui, Lau, & Baldwin [33], have used a variant of LDA to detect languages in multilingual documents.

Similarly, topic modelling has garnered significant importance in political science and rhetoric analysis [29]. Balasubramanyan, Cohen, Pierce, & Redlawsk [34], have used an LDA-based topic modelling approach to investigate reactions of different political communities to the same news. Song, Kim, & Jeong, [35] have used topic modelling on twitter dataset to analyse the socio-political landscape of the 2012 Korean Presidential Election. In Chen, Zhu, Kifer, & Lee [36], the authors have used an LDA model to reduce the size of auto-discovered latent opinions, words and topics in political standpoints between Republicans and Democrats in the US. German National Elections since 1990 was analysed using an extension of LDA (LogicLDA and Labeled LDA) that explored multi-dimensionality of political documents, pushing the limits of content analysis in the social sciences [37]. A detailed explanation of topic modelling choices for political content analysis can be found in [29].

Yano, Cohen, & Smith [38], have used topic modelling using a geographic LDA (LGTA, Latent Geographical Topic Analysis) to extract geographical information in online political blogs. A multi-scalar LDA model was used by Tang et al. [39], to cluster very high-resolution panchromatic satellite images. More recently, LDA was used as a means of data-driven geotopic detection of urban emergencies for natural hazards, manmade disasters and other emergencies [40]. Topic modelling is now being used to map consumer sentiments and understanding their behavioural choices on a geographical scale for enhancing business potential [41], [42]. Critical environmental and social problems are now being identified using twitter-based geo-topics that provide rich knowledge of important events (e.g., cultural activities, political campaigns, accidents, crisis) at an urban scale. Yao & Wang [43], have used a dynamic topic modelling approach to identify urban crisis in US cities that had crucial implications in sustainable city planning and policymaking.

Although the success of LDA-based topic modelling applications in sociology, history, political science and linguistics, topic models are known to suffer from some conceptual and practical problems, like a lack of justification of Bayesian priors, divergences with statistical characteristics of original texts and the imprecision in choosing the relevant number of topics [44]. These causes ontological and empirical biases. Recent efforts in solving these problems refer to the cross-fertilization between multiple fields through mixed-method or multi-method interdisciplinary approaches [44]. Baumer et al. [13], compared the grounded theoretic approach with topic modelling to compare interpretive social sciences (grounded theory) with statistical machine learning (topic modelling). Their results have shown that two analyses produce similar results with field-specific drawbacks. However, their comparison suggested that novelty of using these techniques lies in a compelling combination of such methods as a complementary entity, rather than a ‘replacement’ of one method. In this study, we do not intend to compare all the available topic models (see [29] for such comparative study), neither contribute to the development of such computational models. Here, we derive a humanities and social science-oriented application framework for deep-narrative analysis using topic modelling technique.

There are a handful of studies on the application of topic modelling in energy research. For example, Jiang, Qiang, & Lin [45], applied topic modelling on a bibliometric dataset on hydropower research. They established 29 topics that described the intellectual architecture of hydropower research and found that an interdisciplinary lens in hydropower research is needed for higher-level policy benefits. The authors also established that topic modelling approach in energy research could provide a new outlook to evidence-based policymaking. More recently, Walker, Chandra, Zhang, & van Witteloostuijn [46] have verified the critical importance of topic modelling-based approaches in deriving evidence for policy design and policymaking in public administration.

## 3 Conceptual framework of the proposed nested deep-narrative analysis methodology

**Fig. 1:**
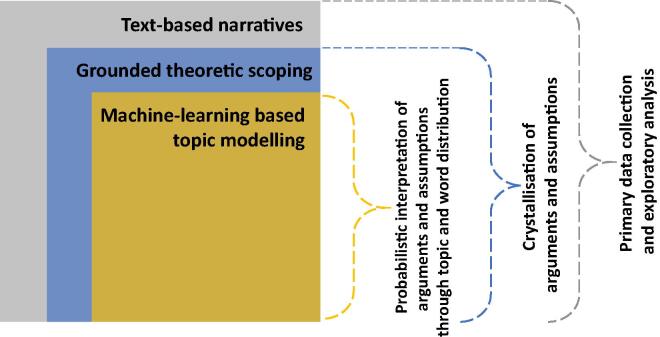
Proposed nested deep-narrative analysis methodological framework based on computational social science and narrative-analysis.

The proposed nested deep-narrative analysis methodology proposed is illustrated in Fig. 1. The framework has three core parts that work in synergy with each other. The nested methodological arrangement is to efficiently extract mutual benefits of topic modelling (TM) and grounded theory (GT). We claim that the introduction of topic modelling in the narrative analysis will not only improve the robustness of the findings for policy application, but it will also reduce the inherent directionality bias. We refer to directionality bias as the biases that are consciously or sub-consciously induced in the interpretation of a narrative-driven research that alter the objective meaning of the findings. These assumptions are coherent with the arguments of Trotter [4] and Bryant & Charmaz [15]. Moreover, from an applied public policy perspective, Walker et al. [46], have argued the need for objective and robust qualitative approaches in evidence-based policymaking. They also point out that the current qualitative methods have a strong directionality quotient (referred to as ‘hegemonic interpretation’, pp 475, [15]) that is sensitive to the interpretability-bias of the researcher, analyst or policymaker.

Our proposed methodology aims to address this limitation by improving the objectiveness of narrative analysis though a nested application of unsupervised machine-learning driven TM while preserving the insight generating capability of narrative by using GT (see Fig. 1). The nested approach proposed here provides a remarkable methodological polyvalence to topic modelling and the grounded theory. It is further discussed with bibliometric evidence in Section 5.1 while establishing the epistemology-fit and the meta-theoretical fit. Topic Modelling (TM) represents the topic detection and clustering algorithms based on the theories of natural language processing and computational linguistics. The Grounded Theory (GT) represents a systematic methodology that conceptualises the latent social patterns and relationships for understanding observed social processes within a specific setting [47].

GT is a structured, yet flexible methodology which is often used to discover systemic information about a little known phenomenon [16], [48]. It can produce or construct an explanatory theory that uncovers the latent process inherent to the area of inquiry [16]. It aims to generate a theory that is grounded in the data [48], and the nested application of TM can add robustness to this process. The current epistemology associated with GT has three distinct methodological genres. The traditional GT generates a conceptual theory that accounts for a pattern of behaviour that is relevant and problematic for those involved. The constructivist GT constructs meaning in relation to the area of inquiry. And the symbolic interactionism is a sociological perspective that relies on the symbolic (subjective) meaning people attribute to the processes of social interaction [48]. We employ the symbolic interactionism of GT in the proposed framework (see Fig. 1).

GT is also referred to as a process by which theory is generated from the analysis of data by the researcher who views the world through their own particular lens [48]. However, its rigour is highly depended on the quality of manual coding of the raw data and the presence of directionality biases in the narrative-collection. We overcome these limitations by the nested application of TM and GT through a deconstructivist approach of breaking down narrative corpus into its fundamental word constituents. The words are then clustered through probability distribution functions (steps of TM) that are used in an interpretivist manner through the lens of GT. In doing so, TM clusters high probable words from a text corpus of narratives, GT aids the analyst in crystallising arguments and insights that statistically inform evidence-based energy policymaking. However, we make it clear that TM should be constructed with specific objectives in mind, rather than statistical optimisation. GT, on the other hand, aids in zooming-through the topics and the narratives and discover insights based on the probability distribution of the topics. A application of our nested framework for energy justice-based policymaking is presented in the part-II of this study [19].

## 4 Methodology

**Fig. 2:**
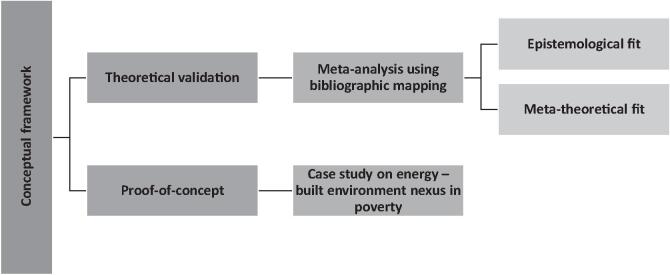
Methodological framework of this study.

This section illustrates the methodological framework of this study that was used to establish a theoretical validity and generate a proof-of-concept of the proposed deep-narrative analysis framework, as discussed in Section 3. The methodological framework of this study consisted of two core parts (see Fig. 2). In the first part, a meta-analysis of published literature was performed to establish the theoretical validity of the proposed framework. The theoretical compatibility was established by expanding two metrics; epistemological fit and meta-theoretical fit (see Fig. 2). In the second part, a proof-of-concept of the proposed framework was derived using a case study. In the case study, externalities associated with demand for energy services in poverty were examined through occupants’ narratives of living in a slum rehabilitation housing in Mumbai, India.

### 4.1 Meta-analysis for theoretical compatibility validation

A meta-analysis for theoretical validation of the deep-narrative analysis framework was performed based on the principles of science mapping [49]. Science mapping is used to represent the body of scientific literature in a tangible form so that one can handle it more effectively [49]. It is used to visualise the scientific landscape at different levels of granularity, i.e. connection between keywords co-occurrences, conceptual relations between groups of articles, similarities between authors, and connections among larger scholarly organizations such as journals and institutions [49], [50].

Using science mapping, we performed a bibliometric network analysis to identify the conceptual structure of narrative-based energy policy research and computational social science-based topic modelling application in the current literature. The conceptual structure maps are used to understand the research landscape and identify what are the most important and the most recent issues. A general science mapping workflow was adopted as per the methodological guidelines of Ayra and Cuccurulo [50] that included bibliographic database extraction (Web of Science or Scopus), data analysis using network theory and data visualisation. The results from the network analysis were used to generate evidence from current literature on the theoretical compatibility of nested application of topic modelling (TM) and narrative analysis using grounded theory (GT) in energy policy research, as per the proposed conceptual framework in Fig. 1.

We followed two lines of argument for evaluating the theoretical compatibility of TM and narrative analysis (using GT) for energy policy applications (see Fig. 2). A meta-theoretical level fit was evaluated to examine congruency between the under-lying conceptual assumptions between TM and narrative analysis. Conceptual structure maps were derived using data reduction technique like multiple correspondence analysis (MCA) and factor analysis. The conceptual structure maps examined the research front of topic modelling and narrative analysis by using the actual content of the documents to construct a similarity measure [50].

At an epistemological level, we argue that the methodological idea behind the nested application of topic modelling and grounded theory fits the analytical process of crystallising arguments and assumptions using narrative analysis. This epistemological fit aided in understanding how topic models generate knowledge about the texts and the words in the corpus for fulling the objectives of ‘deep-narrative’ analysis. A similar line of argument was used by Jacobs and Tschotschel [25] for theoretical compatibility validation of their combined application of topic modelling and discourse analysis in qualitative research.

**Fig. 3:**
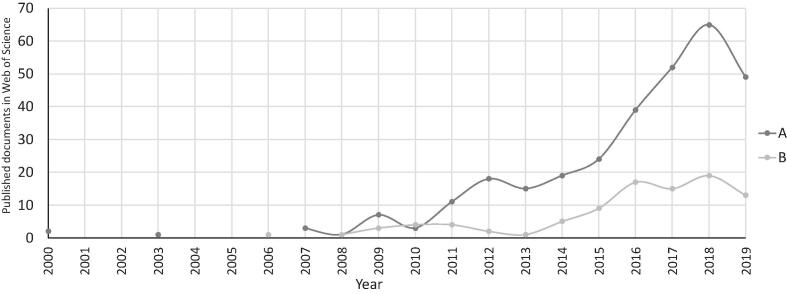
Published documents in Web of Science (WoS) database for the searched keyword set A: ‘energy policy’ & ‘narratives’ (n = 311); and keyword set B: ‘computational social science’ & ‘topic modelling’ (n = 94) between 2000 and 2019.

The Web of Science (WoS) bibliometric database was used as the primary data source for the science mapping. A complete bibliometric extraction was conducted that included ‘title’, ‘abstract’, ‘keywords’ and ‘journal keywords (keyword plus)’ which were used for the factor analysis. The WoS database showed 311 published documents between 2000 and 2019 on the topics ‘energy policy’ and ‘narratives’, referred to as ‘keyword set A’. Similarly, 94 papers were found between 2000 and 2019 on the topics ‘computational social sciences’ and ‘topic modelling’, referred to as ‘keyword set B’ (see Fig. 3). The factor analysis was performed using *FactorMineR* [51] and *factoextra* [52] libraries in R. And the data visualisations were performed using the *ggplot2* [53] and *igraph* [54] R-libraries.

While science mapping represented the data-driven method of establishing the theoretical fit between GT and TM. A detailed rationale on the methodological polyvalence of GT and TM was also established based on the properties and complementarity of both methods.

### 4.2 Case study: deep-narrative analysis for examining externalities associated with energy services in slum rehabilitation housing in Mumbai, India

We present a case study on the application of deep-narrative analysis on understanding energy externalities in poverty. Slum rehabilitation housing (SRH) in Mumbai, India was chosen as the study area, where rising electricity bills are a significant concern [55], [56]. SRH is a slum rehabilitation strategy in India where slum dwellers are rehabilitated to permanent government-sponsored housing. It is a part of the poverty alleviation efforts of the Government of India [57]. The demographics of the SRH understudy lie in an extreme low to low-income categories with informal employment as the primary occupation [58].

From an energy policy perspective, recent studies have shown that the households living in the SRH are pushed into energy poverty as occupants are unable to pay high electricity bills [55], [56]. It was reported that the households are often billed up to 40% of their monthly income which remain significant distress in the SRH under study [55]. The reasons behind rising electricity bills in the SRH are still not clear. However, the literature showed that a shift in energy practices due to a change in their built environment could be a significant factor [56]. The energy practices were shifted because slum dwellers were moved from horizontal slums to a high-rise vertical built form (the SRH) that changed their social organisation [56]. The change of this social organisation is linked to the rise in energy intensity of the households. Besides, the aspiration of the occupants in owning specific appliances in their ‘permanent’ house further illustrated an increase in energy intensity [56], [59]. The aspirations motivated by the change of built environment redefined occupants’ demand for comfort and convenience as cultural energy services [60]. Therefore, we hypothesised that external characteristics associated with the built environment are influencing more significant electricity usage in households, resulting in higher electricity bills.

To investigate this hypothesis, we conducted a focussed group discussion (FGD) with the female household members of the SRH to understand the injustices associated with the current SRH built environment that may have energy poverty implications. Female occupants were specifically chosen because it is common for women to stay at home while the men go to work in the SRH community [59]. Thus, it was assumed that they interact the most with the built environment as they spend the highest amount of time in the SRH. Besides, the female members often organise themselves in self-help groups for built environment maintenance and income-generating activities [55], [59].

The FGD was conducted as per the best practice guidelines of [61]. Informal interviews were conducted to extract information on the energy poverty externalities in poverty in the SRH built environment (see Table 1). The FGD participants were recruited by us asking a local contact who had previously worked with us. The local contacted acted as the FGD moderator. We asked the moderator to bring along some of her friends to the FGD. This method was employed so that a familiar trust was built during the interview process. A total of 11 participants (P1 to P11) joined the interview that lasted for an hour. The primary occupation of the participants was ‘housewife’; however, they all were members of a self-help group called ‘Mahila Milan’. Out of 11 participants, seven were middle school dropouts, while four never attended formal schooling. All participants of the FG spoke the same mother tongue ‘Hindi’. The moderator of the FG was proficient in bilingual translation, who transcribed the interview from Hindi – English. Verbal consent was obtained from each participant before the start of FGD.

**Table 1:** FGD points to explore energy-built environment nexus in slum rehabilitation housing in Mumbai, India.

Energy services	Appliance ownership
	Access to information and communication technology (ICT) devices
	Devices for comfort, cleanliness and convenience
	Cooking energy (clean fuel or firewood)

Built environment services	Access to natural ventilation and its constraints
	Window opening and closing schedule
	Indoor air quality and thermal comfort perception
	Access to open spaces and playgrounds for kids
	Quality of life as compared to horizontal slums

Energy Economics	Energy bills and the possible causes of high energy bills
	Distresses concerning higher energy bills
	Availability of subsidy and appliance repair services

#### 4.2.1 Processing the transcript of the focussed group discussion for deep-narrative analysis

Based on the proposed nested framework in Section 3, the transcript of the focussed group discussion (FGD) was processed in the following stages. The first stage was the preparation of the textual transcript as a primary data object or corpus for topic modelling. The text transcript had a word/term count of about 9000 words that formed the primary data corpus for topic modelling. It was a pre-processing stage where the transcript was cleaned by removing all the stop words (e.g., articles, such as “a,” “an,” and “the,” and prepositions, such as “of,” “by,” and “from”), numbers, and punctuation characters and converted the text to lowercase in the corpora. This process is called lemmatisation [62]. This process also converts the grammatical form of a word into the base or dictionary form (known as Lemma) [62].

The second stage involved performing of topic modelling using Latent Dirichlet Allocation (LDA) algorithm (see Section 2.2 for more information on LDA applications). The lemmatised corpora of the transcript were converted into a document-term-matrix (DTM) using the *tidytext* package [63] in R programming language. The DTM was further calibrated as per the *tidydata* rules (see chapter 1, [64]). We iteratively defined the number of topics based on our judgement and the *ldatuning* package in R [65]. A similar approach in topic model determination was also adopted by [46], [66]. Finally, we used the R-package *topicmodels* to fit the LDA model [67].

The mathematical foundation for the LDA was adopted from Blei, Ng and Jordan [68], see Eq. (1).

(1) $$p\left(D|\alpha ,\beta \right)=\prod_{d=1}^{M}\int p\left(\theta_{d}|\alpha \right)\left(\prod_{n=1}^{N_{d}}\sum_{z_{\mathrm{dn}}}p\left(z_{\mathrm{dn}}|\theta_{d}\right)p\left(w_{\mathrm{dn}}|z_{dn\nu }\beta \right)\right)d\theta_{d}$$

An LDA-driven TM is an unsupervised machine learning (ML) technique that has three hyperparameters (‘α’, ‘β’ and ‘γ’,) to control document and topic similarities. Alpha (α) and beta (β) are crucial in assigning word and topic distribution over the corpus document (see Eq. (1)). Each sentence in the text corpus of ~9000 words was treated as a unique document using the DTM format.

A total of 311 documents were created in the DTM. It is a widely-used methodology for TM, and the cross-validation measures determine its reliability [42], [66], [69], [70]. The third hyperparameter, gamma (γ) that controls the number of topics the algorithm will detect has to be set while implementing LDA. Gamma (γ) is crucial for TM since LDA cannot decide on the number of topics by itself (see, no γ in Eq. (1)). It is the most distinctive feature behind unsupervised TM versus supervised text classification techniques commonly used in qualitative data analysis tools (like NVivo, ATLAS.ti, MAXQDA, etcetera). The unsupervised nature of TM reduces the directionality bias that may get embedded in supervised text classification (as discussed in Section 1). In this study, we determined the appropriate number of topics using the *ldatuning* algorithm, as mentioned above.

The third step included visualisation using the *ggplot2* package in R [53]. Based on the high probability words discovered through the topic models, these words are further expanded using grounded theory to support the narratives on externalities associated with the built environment – energy service nexus in slum rehabilitation housing. Thus, in this manner, we provided a proof-of-concept of deep narrative analysis using the nested application of topic modelling and narrative analysis using grounded theory.

### 4.3 Performing the nested deep-narrative analysis

**Fig. 4:**
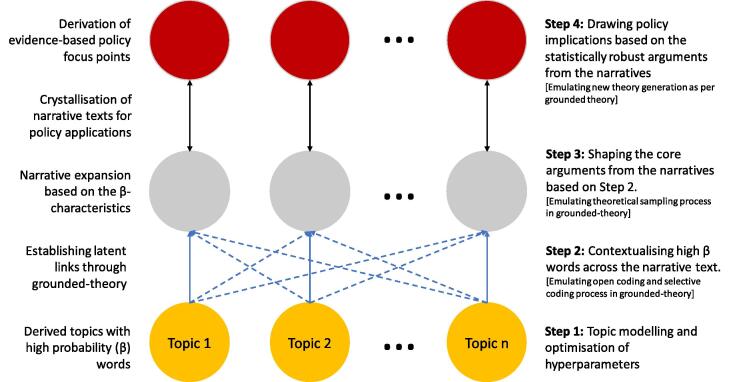
The deep-narrative analysis process.

The four-step process flow for the nested deep-narrative analysis is illustrated in Fig. 4. The results from topic modelling (TM) was used as the starting point for the deep-narrative analysis. The discovered topic and word clusters from TM established the probabilistic (β) background for investigating the narratives (see step – 1, Fig. 4). Based on the high β values of the words in each of the topics were contextualised with the original narrative text. This contextualisation was done using a grounded-theoretic (GT) approach, such that high-β words acted as a surrogate for ‘open’ and ‘selective’ coding process of GT-based research [48]. The contextualisation step intrinsically also linked the latent factors in the narrative data that will enable in-depth understanding of the built environment – energy nexus in poverty in the slum rehabilitation housing in Mumbai. Thus, the step-2 (see Fig. 4) generated our own probabilistic ‘code system’ based on the narrative texts.

However, unlike qualitative data analysis, this code system has an unsupervised probabilistic background. It not only added statistical signification to it, the unsupervised nature of TM also eliminated the directional biases. The code system was now used to perform ‘theoretical sampling’ as per GT (see step 3, Fig. 4). The theoretical sampling, now supported by β-values, was used to develop the emerging theory and elaborate on the main categories constituting the narratives (see step 4). In this study, the derived grounded-theories were used to derive grounded-policy focus points. These focus points are not only independent of directionality bias but also has a degree of certainty due to its statistical foundation.

## 5 Results and discussion

### 5.1 Theoretical validation of the proposed framework

**Fig. 5:**
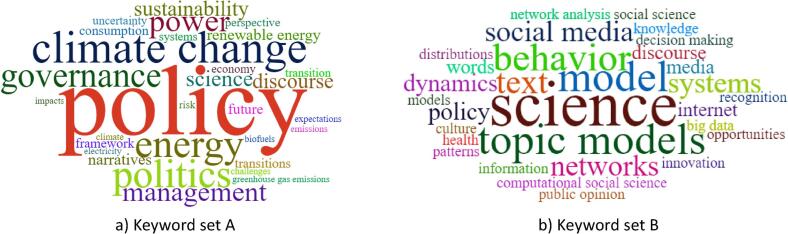
Word-clouds illustrating high frequency words in the WoS database for search keyword set A: energy policy and narrative (n = 311), and search keyword set B: computational social science and topic modelling (n = 94).

The common words in the keyword set A (‘energy policy’ & ‘narrative’) and B (‘computational social science’ & ‘topic modelling’) are illustrated in Fig. 5. It shows that the use of narratives in energy policy has greater use in governance, politics, management, uncertainty analysis and perspective building towards sustainability (see Fig. 5a). It further shows science mapping-based exploratory evidence of the policy applications of topic modelling through public opinion and discourse analysis (see Fig. 5b).

#### 5.1.1 Meta-theoretical fit of the proposed framework

The meta-theoretical fit was used as a line of argument for evaluating the theoretical compatibility of the nested application of topic modelling (TM) and grounded theory (GT) for narrative analysis (see Fig. 2). We termed this nested application as ‘deep narrative-analysis’ (see Section 3 and Fig. 1). The parameter of the meta-theoretical fit was used to examine how an analyst can subjectively and reflexively interpret the derived topic models as foundational elements for deep-narrative analysis. It is the analyst’s task to interpret the meaning of a topic based on how it ranks terms and how it relates to other topics. Thus, from a meta-theoretical fit assessment, our proposed nested application should retain the ‘crystallising’ capability of narrative analysis for arguments and assumptions in a document.

In TMs, the meanings of a word are topic-specific and based on the other words that appear in the topics in which it features prominently [25]. This prominence of topics or words is represented through probability scores in TM. By leveraging these scores, an analyst can employ GT with a higher degree of confidence to connect the hidden dots between the words and the topics, that can explain a broader process or phenomenon. These arguments demonstrate that TM and GT have robust theoretical compatibility for deep narrative analysis applications.

Besides, the nested application of these methods aligns well with the insight generation and argument crystallisation capability of narrative-analysis in policy research. TM explicitly models ‘polysemy’, i.e., the coexistence of multiple possible meaning for a word or phrase [71]. It traces the multiplicity of contexts of every word in the text corpus and assigns a probability value. The GT is inductive, and it aids in linking the latent connections between socio-cultural processes that are sensitive to policy variables (in energy research and social science context) [15]. Moreover, TM induces the quality of directness in the interpretation, which adds robustness to the GT-based narrative analysis.

In TM, the topics themselves obtain their meaning through i) the inter-relationship between words in a cluster of topics; ii) inter-cluster meaning of words between the topics, and iii) through frequent co-occurrences with other topics. Further meaning is derived by linking words to multiple topics, indicating the relationality feature of TM [25]. Besides, GT strengthens the relationality to develop critical insight for policy applications [15]. Thus, enabling an analyst to derive quantifiable evidence from narratives analysis.

**Fig. 6:**
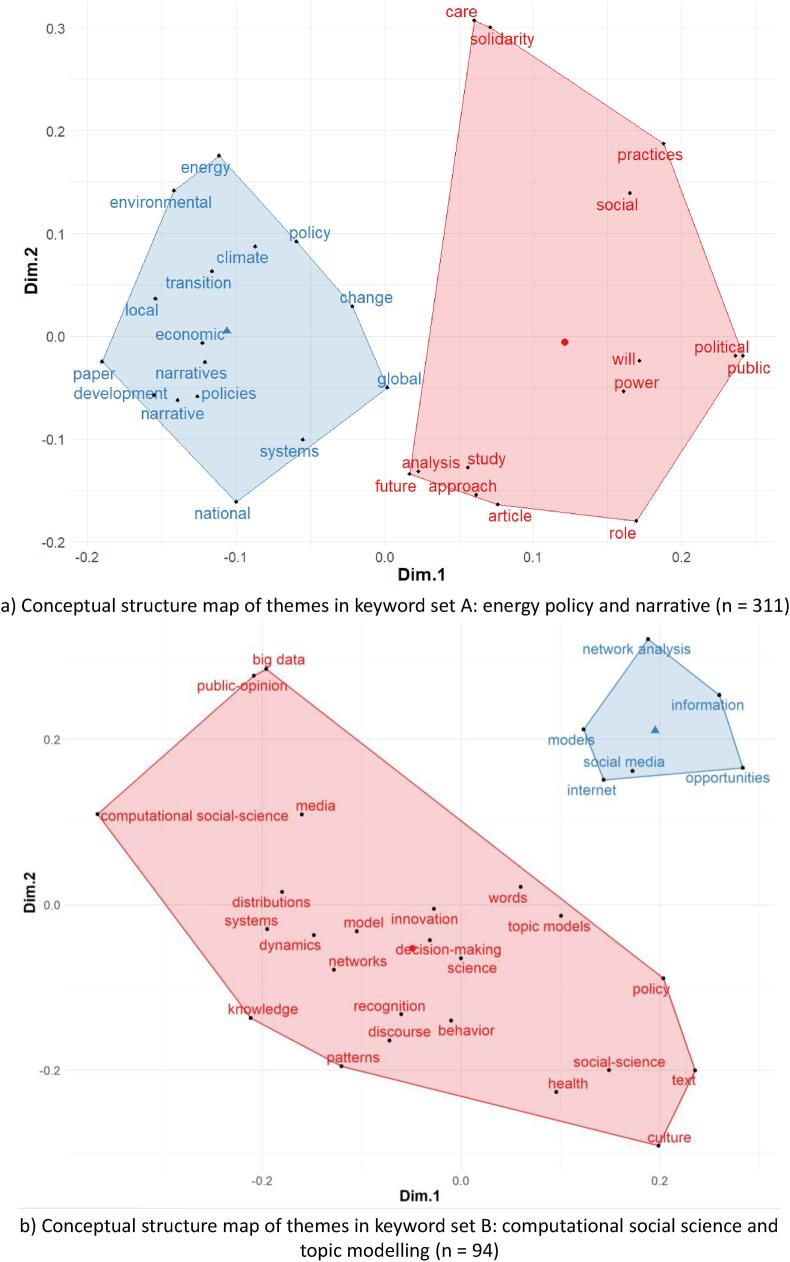
Conceptual structure of keyword set A and set B through science mapping to show epistemological fit.

In TM, topics are interpreted as frames, themes, et cetera, but that the most appropriate interpretation depends on how the method is used by the analyst [25]. We expand on this statement using science mapping to examine the commonality and cross-fertilisation between keyword set A and B, respectively. A higher degree of cross-fertilisation and commonality of concepts between TM and narrative-analysis (using GT) would indicate a methodological polyvalence [25]. The methodological polyvalence would mean that our proposed deep-narrative analysis approach can be applied to a broad scope of energy policy and social science research problems. The conceptual structure maps in Fig. 6 illustrate the cross-fertilisation and commonality of the keyword set A and B, respectively.

The blue and red clusters show the cross-fertilisation of themes in current published literature in the respective keyword sets (see Fig. 4). For Fig. 6a, the keyword set A represents conceptual structures of the published literature on energy policy and narrative analysis. It shows that the cross-fertilisation was in the application of narrative analysis in energy and climate policy research by using public deliberations and opinions as to the primary data source (see Fig. 6a). Similarly, for keyword set B, the red and blue clusters represent the conceptual structure of the current literature on computational social science and topic modelling applications. The cross-fertilisation in keyword set B shows the use of topic modelling in ‘decision-making’, ‘discourse behaviour’, ‘policy’, ‘text’ analysis (see Fig. 6b). Moreover, the blue and the red clusters across Fig. 6a and b show conceptual commonalities between keyword set A and B. The cross-fertilisations of methods and the commonalities show the meta-theoretical fit of TM and GT for nested application in policy research.

We maintain a caution that the use of a qualitative method like narrative analysis using GT with TM should be constructed with specific research objective in mind, rather than with statistical optimisation. For example, in our proposed nested methodology (see Fig. 1), we use TM with GT for deep-narrative analysis such that GT aids in refining the parameters of the model interpretation to facilitate the best possible answer to those research questions. It would mean building the topic models in a way that answers the research questions, rather than adapting research questions so that they can be answered through TM. The later remains a limitation in the current application paradigm of computational social science research for public policy applications [25], [29], [72].

#### 5.1.2 Epistemological fit of the proposed methodology

The second line of argument for theoretical validation of the proposed framework was a science mapping-based investigation of the epistemologies of Topic Modelling (TM) and narrative analysis (using GT) in energy policy research. The commonalities in the conceptual structure maps in Fig. 6 illustrates a degree of coherentism [73] that indicates an internal validity between the applied concepts of TM and GT. However, it is impossible to establish external validity between TM and narrative analysis, as they have different epistemological grounds (also pointed out by [25] for TM and discourse analysis).

Epistemologically, TM is an unsupervised method and inherently inductive approach. TM purely represents the pattern of language use within the text corpus, independent of externalities associated with the text. It adheres to the epistemology of the text corpus. However, GT-based narrative analysis employs a supervised application, where the analyst pre-defines categories or scales of the narratives understudy in a deductive fashion. It can induce directionality bias in the interpretation of the results (as discussed in Section 1 and Section 2.1). Our proposed nested methodology can aid in reducing this directionality bias due to the unsupervised and inductive nature of TM. These characteristics of TM strengthen its applicability in policy research, as illustrated by the conceptual structures in Fig. 6b.

It is the analyst’s task to interpret and make sense of TM and generate valuable insights based on the semantic relations and crystallised arguments from the narratives. The subjective input of the analyst thus continues to play a vital role and cannot be replaced by TM algorithms for ‘good’ policy analysis. TM enables the analyst to effectively condense or transform large corpus of narrative texts upon which the analysis realises the analysis itself guided by GT (as per the nested methodology, Fig. 1). The conceptual structures in Fig. 6 further illustrate the parallel epistemologies that both narrative-analysis and TM represent in the scope of policy analysis. It essentially demonstrates the type of questions that can be typically investigated using our proposed methodology. For instance, the red clusters in Fig. 6a and b, respectively, thematically hints towards the meaning-generating characteristic of TM and narrative analysis. Words like ‘discourse’, ‘practice’, ‘behaviour’, ‘public opinion’, ‘knowledge’, ‘distribution’ across set A and B represents the application-specific commonality of TM and narrative analysis for energy policy application. As mentioned above, these commonalities necessarily establish the internal conceptual validity of TM and narrative analysis (using GT).

The arguments we developed here concerning the theoretical compatibility of TM and GT-driven narrative analysis centres around the cross-fertilisation and the meaning-generating capability of both the methods in an energy policy research context.

The science mapping and the conceptual structure between the two methods (see Fig. 6), illustrated the complementary application spaces in energy policy research. This data-driven approach established the theoretical compatibility from a policy applied perspective, thus proved the ‘applied’ scope of this paper. In Table 2, we further elaborate on the theoretical fit based on the properties of TM and GT and the synergistic benefits it offers to deep-narrative analysis.

**Table 2:** Theoretical complementarity of using TM and GT for deep-narrative analysis.


	Topic Modelling	Grounded Theory
Properties and epistemologies	• A quantitative approach from natural language processing; • Positivist approach; • Unsupervised process;	• A qualitative approach from interpretive social science; • Interpretivist approach; • Human reading and supervision required;

Methodological strength	• Purely data-driven; • Requires no human supervision to identify and define topics; • Rigour is established through probability distribution over a vocabulary; • Identifies critical themes from large corpus of documents ranging from thousands to millions of textual data points. • Enables generalisability through data-driven reasoning	• Generate rich, thick, descriptions; • Construct theories by interlinking latent social processes; • Provides comprehensiveness across the diverse sociological orientations by making interpretive methods more codified, legible and legitimate; • Rigour is established through confirmation of findings, either from informants or from other researchers; • Provide a common interleaving of data collection, analysis and theorization

Sources of bias	• Encoding and Empirical bias; • Algorithmic bias; • Over predictability; • Epistemic attachment to the object of research	• Directionality and interpretivist bias; • Lack of robust verifiability metrics • Significant amount of human time and energy required

Inferences from science mapping	• Energy policy application space to understand social practices, political and public opinion, innovation models, discourse and behavioural analysis, etcetera (see Fig. 6); • Conceptual structure from current bibliometric dataset showed high intellectual commonalities with the narrative analysis for energy policy applications (as discussed in Section 5.1)	• Widely used in narrative analysis in energy and climate policy applications; • The conceptual structure showed its application space in improving and contextualising semantic relations in text datasets, a much-needed element for drawing targeted policy implications.

Complementarity from nested application	• The nested application limits the biases and creates a complimentary scope for both positivist and interpretivist policy research; • The nested application enhances the synergistic capability of both the methods such that topic modelling removes the need for open and selective coding and theoretical sampling. Thus, reducing the sources of bias in grounded-theoretical research. • While at the same time, grounded theory aids in contextualising the discovered topics and its highly probable words from the textual corpus. It the nested application process, explained in detail in Section 4.3. • It brings in data-driven capabilities of machine learning while simultaneously maintaining the linguistic, contextual and interpretive insights of human reading.	

### 5.2 Case study: energy poverty and built environment externalities in slum rehabilitation housing

**Fig. 7:**
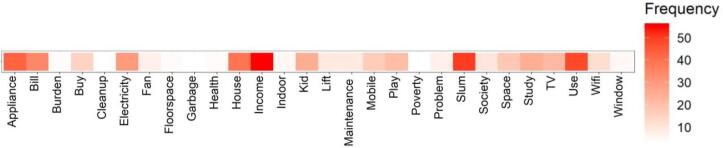
High frequency words from the FGD transcript (n = ~9000).

The narratives collected through FGD using the questionnaire themes in Table 1, examined the role of built environment parameters in energy service demand and quality of life in poverty. The exploratory indications provided through high-frequency words in Fig. 7 indicated some interconnected relationship between income, appliance ownership, electricity bills, housing standards and the built environment elements like windows and open spaces, garbage disposal (see Fig. 7). Besides, the quality of life indicators was in the form of effect on kids’ study and play routines, and the health and well-being of the occupants (see Fig. 7).

**Fig. 8:**
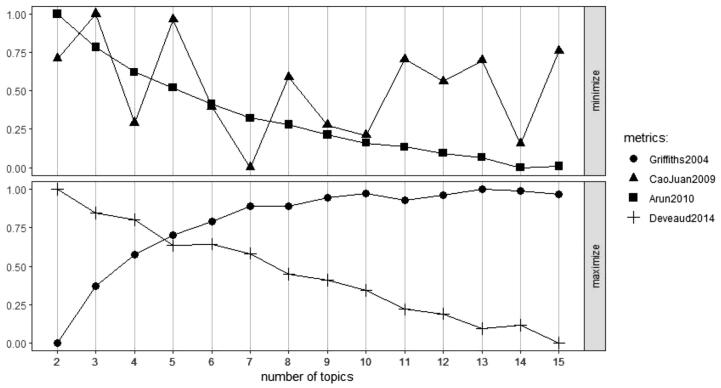
LDA tuning convergence for topic approximation.

Seven topic clusters were found to extract the most relevant information from the FGD narratives (see Table 3), which was verified using the LDAtuning algorithm as well. The CaoJuan2009 metric in the LDAtuning reached a global minimum at seven topics, whereas Griffiths2004 metric showed a global maximum at ‘13′ topics (see Fig. 8). It was observed that specific terms began to repeat frequently beyond seven topic clusters which were inducing redundancies in meaning. Therefore, we decided on seven topics (see Table 3).

**Table 3:** Discovered topics based on the narratives on built environment – energy nexus in slum rehabilitation housing.

Topic 1	Prob(β)	Topic 4	Prob(β)	Topic 7	Prob(β)
Bill	0.080	Appliance	0.070	Fan	0.065
Irregular	0.025	Want	0.038	Refrigerator	0.021
Month	0.022	House	0.029	Food	0.020
Electricity	0.019	Purchase	0.022	Time	0.031
High	0.018	New	0.020	Cook	0.020

Topic 2	Prob(β)	Topic 5	Prob(β)
Lift	0.039	Space	0.026
Lack	0.030	Lack	0.021
Repair	0.030	Park	0.018
Watch	0.035	People	0.019
TV	0.028	Life	0.016

Topic 3	Prob(β)	Topic 6	Prob(β)
Play	0.050	Window	0.045
Kid	0.048	Close	0.035
Lack	0.020	House	0.028
Space	0.020	Garbage	0.017
Corridor	0.018	Space	0.012

Following the proposed nested deep-narrative analysis trajectory (see Fig. 1), the topics discovered in Table 3 are now expanded to reveal latent social processes that can outline externalities associated with energy usage in the slum rehabilitation housing (SRH) built environment. High probability (β) terms like the bill (β = 0.080), irregular (β = 0.025) and month (β = 0.025) have critical significance in the narratives from the SRH because irregular high electricity bills that get piled up for months is significant distress in the SRH. Occupants mentioned that sometimes they get bills after three months which is almost as their monthly household income (~60–90 USD), whereas sometimes they get the same amount in a single month.

*“… I get huge variation in monthly bills, sometimes we get Rs 1300 (~18 USD) and sometimes Rs 2000 (~26 USD) … it is usually on the higher side … The distribution company changes fast in area and so are the bills*…” (P3)

The above narrative from one of the participants (P3) revealed that a possible reason for irregular bills could be linked to the frequent change of electricity distribution companies in the study area. This narrative on irregular and high electricity bill in the SRH echoed from other participants (P6 and P7) as well,

“… *bills were very high due to transfer from Reliance to Adani Power Company in this area. We used to get bills in the range of Rs 300–500 (~4–7 USD) but during that month the average bills were in the range of Rs. 2000–5000 (~26–67 USD) …. the bills were 15 days late as well. Many people from the society complained of getting bills as high as Rs. 10,000 (~133 USD …. it is more than the monthly salary of many families living here*…” (P6)

“… *since the Adani Power Company took the charge of electricity distribution in this area, there are problems with the bill …. the bills are not regular and always high*…” (P7)

Thus, administrative lags due to the frequent change of distribution companies in the SRH can be regarded as a critical energy service externality that influences higher energy bills and induces distress in the households.

Topic 2 revealed another critical energy service externality in the SRH built environment that is related to the lack of repair and maintenance of the elevators (see Table 3). Without access to elevators, mobility of most of the occupants got restricted that disrupted their strong social network in the community. This disruption has a substantial but latent cost on the well-being of the occupants. Similar findings on SRH built environment effects on the well-being were also reported in [55], [74].

“… *I am losing contact with my previous slum community members…. some of them stay on the 7th floor while some are on the 4th floor and some on the ground floor …. we do not have a common socialising space and the lifts are always broken people …. living on the top floors do not usually come down for socialising unless it is something important* …” (P11)

“… *I stay on the second floor and I have knee problems …. even if my friends on the 7 floor invites me in the evening, I cannot climb five floors and I miss all the social gathering…. the lifts don’t work, so I am totally stuck in my second-floor living*….” (P4)

An energy implication of the lack of mobility in the SRH built environment due to broken lifts is that physical socialising is declining. In contrast, internet socialising and TV viewing hours are increasing. Such changes in energy practices are also related to the rise in household electricity bills (also reported in [56]). Thus, topic 2 (see Table 3) crystallised the externalities associated with the lack and maintenance of lifts in the SRH built environment that is indirectly influencing occupants to shift to energy-intensive practices.

Similarly, Topic 3 and Topic 5 in Table 3, illustrates the critical terms on the lack of public and community spaces that have adverse impacts on the health and well-being of the occupants especially kids (P5). A participant (P10) revealed that kids do not have a place to play, and they are forced to stay indoors and watch TV or play games on mobile phones. It is the energy externality associated with the lack of playground or parks in the study area. Parents do not allow kids to play on the ground floor as these zones are not safe due to alcohol and drug abuse (P3).

“…*the kids are the biggest sufferers here; they do not have any place to play. They cannot play on the staircases because they can fall hurt themselves …. the occupants living next to the staircases do not let them play because of the playing noises* ….” (P10)

“.… *they don’t have playgrounds …. they cannot play in corridors …. We never allow them to play in the ground floors, because if they go there 100% chances are that they will get addicted to either drugs or alcohol* ….” (P3)

“… *the general health of the kids in the neighbourhood is going down …. teachers tell us in parents-teacher meetings that allow your kids to play daily for at least 2 h, but they don’t understand that we don’t have any safe space here for the kids to play …. Even doctors tell us that they are getting weaker because of lack of physical activities* ….” (P5)

“… *no playground and we are forced to keep our children busy with cartoons in the TV or mobile games… almost all the kids in this society has prescription glasses* ….” (P8)

Topic 4 and Topic 7 discovered terms associated with the demand for energy services through appliance ownership in the SRH (see Table 3). Words like purchase (β = 0.022), new (β = 0.020) and want (β = 0.038) illustrated the aspirational and want-based element of purchasing appliance on moving into the SRH from the horizontal slum. Fulfilling these aspirations also add to the economic distress in the SRH, as more appliance use increases the energy intensity of the households [56], [60]. Moreover, occupants purchase these appliances using money borrowed from informal money lenders or friends (P3 and P11). This debt adds to the economic distress of living in the SRH.

“…. *no, we can never afford to pay out of pocket…. we borrowed the money from a local money lender…. Some people bought appliances on monthly instalments from the stores itself* ….” (P3)

“…. *we just had a fan and a light bulb when we shifted here from the slums… we had to purchase all the appliances by taking loans…. Now we have borrowed money from money lenders to repay the loans* ….” (P12)

Besides, appliances like refrigerators and washing machines were purchased for the greater convenience of living in the SRH (also reported in [60]). Few occupants saved time from using such appliances and used the extra time for income-generating activities. For example, one of the participants (P2) uses refrigerator not only for storing food but also to store flowers that she can sell in the morning market.

“…. *most common items that are stored in the refrigerators are vegetables, water and item related to subsistence activities like flowers, garlands and home-made dairy products like curds, paneer etc* ….” (P2)

**Fig. 9:**
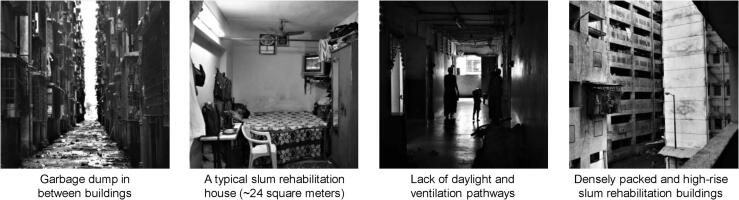
Characteristics of the study area: Slum Rehabilitation Housing, Mumbai, India.

Topic 6 (see Table 3) illustrated some critical aspect of the SRH design elements that have a significant influence on the energy use behaviour of the occupants. For example, the terms window (β = 0.045) and close (β = 0.035) referred to the common practice of keeping windows closed all the time due to lack of hygiene in the built environment. Besides, occupants dump garbage in between the buildings that make it a breeding ground for rats and insects (see Fig. 9).

“…. *the design of the house is very faulty …. the builder has provided with sliding windows which always block half of the opening …. plus, we have such small rooms, the rest of the windows are blocked by our storage items…. Pests are also a big menace here*….” (P8)

“ …. *the problem of rats is huge here …. if we keep our windows and doors open, they would enter the house and eat up all the stored grains …. I had lost 2 kg of rice last month to rats …. we seal the house completely by covering doors and windows which makes it very hot and uncomfortable…. But we do not much choice*….” (P2)

“… *the rooms are small… we have lots of thieves in the complex …. and so many rats … we have to keep the door and windows closed for all the time which makes our room suffocated*….” (P11)

The energy implications on keeping the windows and doors closed for almost 24 h is on poor thermal comfort and indoor air quality ranges (also reported in [75]). The rooms become stuffy, and occupants must use additional fans or air conditioners to keep the room cooler at an added economic cost. Thus, the poor design, lack of hygiene and safety of the SRH built environment are the energy externalities that indirectly affect higher electricity bills.

The deep-narrative analysis performed using the seven topic models (see Table 3) revealed the latent factors that contribute to higher energy bills and its associated distress in the study area. These underlying factors were further statistically explained using the probability values of specific terms that were critical to the socio-cultural construct of the slum rehabilitation housing under study. Understanding and implementing the particular requirements of such socio-cultural constructs can aid in better policymaking, especially in vulnerable and low-income communities. Thus, using the deep-narrative analysis, we could highlight the critical externalities of energy-built environment interaction that lead to high energy bills in SRH.

**Fig. 10:**
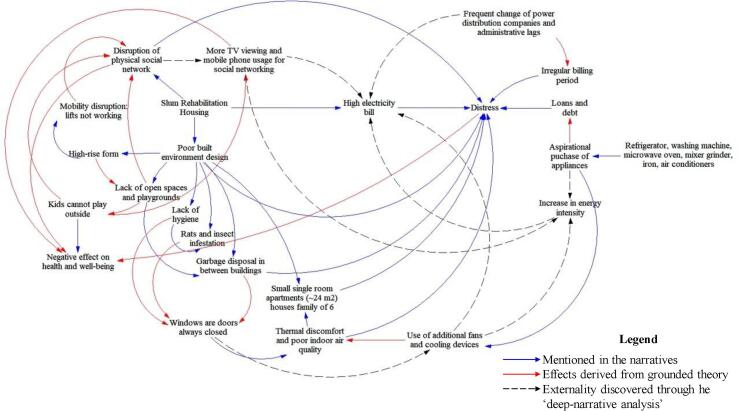
Energy service externality map in the slum rehabilitation housing under study.

A generalised representation of derived externalities is illustrated in Fig. 10. The blue and red arrows represent the narrative-based observations and effect, respectively. The dotted arrows show the latent externalities as derived through the deep-narrative analysis. It is not uncommon that such latent links could also be derived through a robust application of qualitative-only grounded theory and narrative analysis. But as mentioned in Table 2, the robustness of such use remains unverifiable. Tt causes a reduced degree of generalisability. From a pure qualitative textual analysis perspective, improving robustness, in this case, would mean increasing the cyclicity of open coding, selective coding and theoretical sampling process in GT, until a reasonable theory is constructed. This is manually intensive and time-consuming. Moreover, the theory construction phase can induce directionality bias. Baumer et al. [13] explicitly called for the development of mixed-method approaches to address this weakness of GT (discussed in detail in Section 2).

Our nested approach provided a probabilistic background for GT to rank words based on the relative similarities across the discovered topics. It further aided in the crystallisation of FGD texts for drawing the latent links (see dotted lines in Fig. 10). Until this stage, it was an unsupervised process that eliminated the possible source of directionality biases, and the probability values (β-values, see Table 3) improved our degree of confidence in connecting the latent processes in significantly less time. Furthermore, the nested application (see Fig. 4) preserved the contextual and interpretive insights from the narrative texts. Thus, enabling a similar elaborate analysis that can be achieved through manual coding using any qualitative data analysis (QDA) tools. It highlights the strength of our nested methodology that it does not intend to replace any existing QDA methods, but instead brings in the mutual benefit of machine learning in qualitative research and policy analysis.

## 6 Concluding remarks

This study presented a novel nested deep-narrative analysis approach using topic modelling and grounded theory for energy policy application. It emerged from the need for a multidisciplinary approach in energy policy research that transcends beyond the ‘technical-only’ feasibility of specific scientific methods to address energy and climate injustice [1]. The intention behind the proposed methodology is to contribute to the state-of-the-art literature and strength the use of text-driven policy analysis tools like narratives, stories and storytelling using computational social sciences. Our proposed methodology does not replace qualitative methods like grounded theory or narrative-analysis, rather enhances its insight generating capability by using Bayesian policy modelling and evidence analysis methods based on the laws of probability. We have argued that the combination of probabilistic models with the grounded theory for narrative-analysis may reduce directionality biases in the interpretation of results in energy policy research. The directionality biases are reduced because the proposed model depends solely on the word-word, word-topic and topic-topic interrelationships, defined by probability distribution functions. It enables the analyst/researcher to build the topic models in a way that answers the research questions, rather than adapting research questions so that they can be answered through topic models. It is expected to enhance the insight generation capability of topic modelling in policy research. Walker at al., [46] argued that the absence of this insight generating ability in the current methods of topic modelling in policy research is a structural limitation.

We examined the theoretical compatibility of both the methods in the nested approach for deep-narrative analysis using science mapping of a bibliographic database to capture the conceptual structure of narrative analysis application in energy policy research and computational social science using topic modelling. The conceptual structure map showed the cross-fertilisation and commonalities of the concepts associated with the narrative analysis and topic modelling. The theoretical compatibility showed that our proposed nested approach could aid the analysis can subjectively and reflexively interpret the derived topic models using a grounded theoretic lens. It not only preserves the ‘crystallising’ capability of narrative analysis for arguments and assumptions in a document, but it also enables the analyst to statistically link the critical terms\topics with the broader context of the arguments. Our theoretical compatibility test showed that the cross-linking capability of topic modelling and narrativity analysis using grounded theory could be established due to the internal epistemological fit of the concepts of these two methods. However, they have different external epistemological backgrounds and cannot be used as a replacement for each other.

Furthermore, we provided a proof-of-concept of the deep-narrative analysis framework by investigating the externalities associated with energy services in slum rehabilitation housing (SRH) in Mumbai, India. The application of the framework revealed latent links between energy use and the built environment in the SRH that influence high energy bill (see Fig. 9). While the focus group discussion of the occupants revealed the problems and the energy culture associated with living in the SRH, but the topics discovered through LDA aided in picking-up critical points in the narratives using probability values. Thus, transforming narratives toward statistical evidence for policy analysis.

In the concluding remark, we want to emphasise on the limitation and implications of our methodology. Not all narrative analysis can or should be done using topic modelling techniques as it deconstructs the text corpus into its barebones. The applicability of topic models is up to the judgement of the analyst a case-by-case basis, assessing the method, the theory and the material at hand. From an ontological perspective, some problems may arise while combining topic modelling with a grounded theory is over-representation of a problem that may bias the results and implications. Therefore, even in the nested application, the analyst must have a clear understanding of the context. Another bias inducing factor in our nested use is the heavy reliance on the topic models to discover critical focus points. It can further dilute the interpretivist nature of the deep-narrative analysis framework.

Some parameters that can induce biases in the interpretation of the results is the lack of interdisciplinary viewpoints. Stressing too much on one aspect of the interpretation may deem the computational part redundant. Besides, relying too much on the probability values to derive critical conclusions can also make the analysis weaker. The analyst should leverage the nested philosophy of the deep-narrative framework and facilitate a balanced interpretivist approach of both topic models and the grounded theory. Ontological bias like epistemic attachment to the object of research can also misinterpret the derived topic models. Such misinterpretation may lead to over-predictability of topics, inducing encoding and empiricist bias. It can influence the understanding of the cause and effect relationships of the analysis. Our methodology does not solve all the challenges associated with either method. Rather, it aids in the development of some of the critical questions which systematically includes qualitative evidence into policymaking through a realist, rationalist and relativist ontological positioning.

We limit our comment on the applicability of the proposed nested model in a small – dataset, like narratives collected through focussed group discussions or personal interviews. Most applications of topic modelling in the state-of-the-art literature is based on big data, whereas narrative is micro-datasets. Sensitive analysis of a big data-driven versus a micro-data driven narrative analysis and policy interpretation remains a gap in the current literature. The case study presented here showed promising results concerning the application of our proposed model in narrative-like micro datasets. We further explore the efficacy of our proposed framework in an energy justice and poverty alleviation context in [19].

## CRediT authorship contribution statement

**Ramit Debnath:** Conceptualization, Methodology, Software, Validation, Formal analysis, Investigation, Data curation, Writing - original draft, Writing - review & editing, Visualization, Project administration, Funding acquisition. **Sarah Darby:** Writing - review & editing. **Ronita Bardhan:** Investigation, Writing - review & editing. **Kamiar Mohaddes:** Writing - review & editing. **Minna Sunikka-Blank:** Supervision, Writing - review & editing.

## Declaration of Competing Interest

The authors declare that they have no known competing financial interests or personal relationships that could have appeared to influence the work reported in this paper.
